# The application of flipped learning to a gross anatomy dissection course

**DOI:** 10.1371/journal.pone.0298067

**Published:** 2024-02-13

**Authors:** Eun-Kyung Chung, Heoncheol Yun, Kwang-Il Nam, Young-Suk Cho, Eui-Ryoung Han

**Affiliations:** 1 Department of Medical Education, Chonnam National University Medical School, Gwangju, South Korea; 2 Institutional Research Center, Chosun University, Gwangju, South Korea; 3 Department of Anatomy, Chonnam National University Medical School, Gwangju, South Korea; AIIMS: All India Institute of Medical Sciences, INDIA

## Abstract

We implemented flipped learning for a gross anatomy dissection course and compared its effects on students’ motivation and academic achievement with those of traditional dissection methods. We invited 142 first-year medical students at Chonnam National University Medical School to participate in this study. All participants engaged in traditional dissection methods in the first part of the study and flipped learning in the latter part. Medical students’ motivation to learn anatomy by cadaveric dissection was measured using the ARCS (Attention, Relevance, Confidence, and Satisfaction) model. Thereafter, all students completed a written examination consisting of 96 multiple-choice questions. The students’ mean motivational score regarding attention was significantly higher in association with flipped learning than with traditional learning. However, the students’ mean motivational scores regarding relevance, confidence, and satisfaction were not significantly different between the methods. Additionally, the mean anatomy practice test score was significantly higher in association with flipped learning than with traditional learning. The students’ motivational scores and anatomy practice test scores associated with flipped learning positively correlated with the extent of learning material completion. The students’ responses indicated that flipped learning helped enhance the learning process, improve time management, reduce confusion during practice, and promote independent practice. The application of flipped learning to a cadaveric dissection course increased individual learning motivation, which improved learning activities both in and out of class, as well as academic achievement.

## Introduction

The flipped classroom is an educational paradigm that inverts traditional elements of lecture and homework in course design [1, 2]. It allows students to initially engage with the learning material before class, thus repurposing class time for active learning exercises in small groups, which facilitates a deeper understanding of the subject matter. Flipped learning is attractive to both students and teachers because it provides pre-class self-regulated learning, in-class active learning with peers, ongoing after-class activities, and assessments aligned to achieve the desired objectives [3]. Teachers can spend their face-to-face time supporting students in deeper learning processes [4]. The flipped classroom is a student-centred learning strategy in which each student is responsible for coming to class with a basic understanding of the material so that he or she can fully participate and engage in class [5]. This emphasis on student-centred active learning fosters students’ motivation to acquire knowledge and enhance skills [6]. In particular, deliberative practices, such as cadaver dissection, require more attention and engagement in the activities to improve performance [3]. The success of flipped learning in cadaveric dissection teaching may depend on students’ undertaking substantial independent out-of-class work [7].

Cadaver dissection plays a formative role in the development of professional competence in medical education. It facilitates learning about the human body, as well as confronting–and developing reasoned and compassionate attitudes toward—death and dying [8, 9]. Dissection helps to build teamwork that requires students to work cooperatively under difficult, emotionally demanding conditions to accomplish their shared goals related to their future profession [10]. However, there is an uneven level of participation within a group because some students are still adapting to the stresses of dissection, and some have not prepared well before dissection sessions [11, 12]. Peer-assisted learning in dissection laboratories is an effective way to encourage student participation and peer group interaction, but the tendency to rely on competent peers often persists [13, 14]. Ultimately, an individual’s attitudes and behaviours are related to motivation [7]. Flipped learning in dissection courses has the potential to enhance the development of self-directed learning as a professional attribute, but few studies have investigated the application of flipped learning to dissection practice [12, 15].

Abeysekera *et al*. proposed that flipped learning pedagogy can increase students’ intrinsic and extrinsic motivation if it creates a sense of competence, autonomy, and relatedness according to self-determination theory [7]. Flipped learning allows students to have the autonomy to study material at their own pace before class, actively learn related content for in-class discussion with peers, and determine their competencies through embedded self-assessment [3, 7]. The benefit of autonomous motivation is positively associated with behavioural persistence, depth of learning, and satisfaction [16]. Therefore, flipped learning has been incorporated into existing peer-assisted learning in cadaver dissection to improve individual engagement and learning outcomes. The first half of the dissection practice was conducted using the traditionally implemented peer-assisted learning method, where student tutors who prepared for the dissection in advance helped peers to learn, with the peer tutors reinforcing their own knowledge through the act of teaching. The second half included the addition of a flipped learning method, in which all students studied video-based materials and solved accompanying quizzes prior to the dissection. This study aimed to compare students’ motivation, out-of-class work, and academic achievement between traditional peer-assisted learning and flipped learning, as applied to cadaver dissection. The research questions were as follows:

1. Are there any significant differences in students’ motivational scores and practice test scores between traditional methods and the flipped learning method?
2. With the flipped learning method, is there any significant variation in students’ motivational scores and practice test scores according to the extent of learning material completion?
3. How satisfied would students be with flipped learning in a gross anatomy dissection course?

## Methods

### Study participants and dissection course

We invited 142 first-year medical students at Chonnam National University Medical School to participate in this study. The study was approved by the Chonnam National University Institutional Review Board (IRB No. 1040198-230418-HR-039-02), and all participants consented to the use of their data. The sample consisted of 108 (76.1%) male students and 34 (23.9%) female students.

All participants had completed a course on gross anatomy as part of the standard curriculum. The dissection component consisted of 16 3-h sessions designed to independently address the back, chest, upper limbs, abdomen, pelvis, lower limbs, head, and neck. During the dissection component, an intact cadaver was allocated to each of the 23 groups of students. Of these, four groups were comprised of seven students each, while the remaining 19 groups each had six students. All participants engaged in traditional learning methods in the first part of the study period—encompassing the back, chest, and upper limbs. The flipped learning method was used for the latter part of the course—encompassing the abdomen, pelvis, lower limbs, head, and neck. A traditional peer-assisted teaching and learning strategy for dissection courses was implemented at the study institution in 2012, and this constituted the traditional learning method investigated in this study [13]. Of the 23 student groups, four groups took the roles of tutors during the other groups’ respective rotations. In other words, twenty-five students in four groups served as tutors, and one or two tutors were assigned to each of the other 19 groups during the dissection course. The roles of students were then switched during subsequent rotations. Tutors dissected intact relevant cadaver parts in advance with the guidance of textbooks and video clips; they could ask questions to faculty members if they faced difficulties. Members of the four tutor groups provided 10-minute explanations of their pre-session dissections to each tutee group before the start of the dissection. These explanations addressed issues, such as important anatomical structures and how to perform specific steps; they also identified the structures that should be carefully manipulated. In the flipped learning method, the dissection course proceeded in the same way as the traditional method; however, before the dissection course, the students were required to study the material covered in individual dissection sessions in advance using e-Anatomy^Ⓡ^ (Panmun Education, Seoul, Korea). This preparation involved engaging with a series of video clips produced by the Korean Society of Anatomy, which included detailed dissection guides. These guides featured explicit learning objectives, the sequence and techniques of dissection, as well as the anatomical structures to be identified for each body part. The video clips ranged in length from 10 to 40 minutes each, and students were given a link to the Learning Management System (LMS) to learn one or two video clips per dissection component.

### Data collection

The medical students’ motivation to learn anatomy by cadaveric dissection was measured using the ARCS (Attention, Relevance, Confidence, and Satisfaction) model [17]. The survey comprises 29 statements, to which students respond using a 5-point Likert scale (5 = very true, 4 = mostly true, 3 = moderately true, 2 = slightly true, 1 = not true). This survey has four categories: Attention (capturing interest and curiosity of learners), Relevance (connecting the instructional content to learner’s academic goals), Confidence (helping students establish positive expectancies for success), and Satisfaction (positive feeling about the learning experience). It contains seven items for Attention, eight items for Relevance, seven items for Confidence, and seven items for Satisfaction. Survey questions were modified after a pre-test targeting 15 second-year students who had completed cadaveric dissection. The students were asked to complete the 29-item paper-based survey twice: once after traditional learning and once after flipped learning. The surveys demonstrated high internal consistency with Cronbach’s alpha values of 0.861 and 0.867, respectively. During the survey on the flipped learning method, the students also completed a self-administered questionnaire to report individual perceptions of the experience provided by flipped learning. Evaluation of student performance in each dissection component was conducted through group presentations of dissection results and individual reports, which were rated on a 3-point scale (3 = exceeded expectations, 2 = met expectations, 1 = did not meet expectations). Immediately after completing the dissection courses, all students completed a written examination that consisted of 96 multiple-choice questions.

### Data analysis

To assess the impact of the teaching methods on student outcomes, we used paired-samples t-tests to compare students’ motivational scores, anatomy practice presentation and report scores, as well as written exam scores between the traditional and flipped learning methods. After stratifying the extent of completion into quartiles, we used one-way analysis of variance (ANOVA) with Tukey post hoc analysis to test for completion-associated differences in students’ motivational scores and anatomy practice test scores related to the flipped learning method. All analyses were performed using SPSS statistics for Windows, version 27 (IBM Corp., Armonk, NY, USA).

## Results

The students’ mean motivational score for the attention subscale was significantly higher in association with the flipped learning method than with traditional learning. However, the students’ mean motivational scores for the relevance, confidence, and satisfaction subscales were not significantly different between the methods. Additionally, the mean anatomy practice test score was significantly higher in association with flipped learning than with traditional learning (Table 1).

**Table 1:** Comparison of students’ motivational scores and anatomy practice scores between the traditional method and flipped learning method.

Category	Traditional method	Flipped learning method	p-value
Motivational scores			
Attention	4.06±0.53	4.11±0.51	0.038
Relevance	4.43±0.44	4.42±0.49	0.660
Confidence	3.62±0.43	3.62±0.45	0.876
Satisfaction	4.02±0.46	4.05±0.49	0.258
Total Score	4.05±0.36	4.07±0.38	0.263
Anatomy practice scores			
Presentation and report	2.06±0.32	2.84±0.38	0.020
Written examination	45.51±11.19	56.52±13.95	<0.001

Unit: mean±SD

Table 2 summarises the ANOVA and Tukey test findings. The ANOVA analysis revealed significant differences in the students’ motivational and practice test scores—associated with flipped learning—according to the extent of learning material completion. Tukey post hoc analysis revealed that students’ motivational scores were significantly higher among students scoring in the top quartile in terms of completion extent compared with those scoring in the bottom two quartiles.

**Table 2:** Differences in students’ motivational scores and anatomy practice scores associated with flipped learning according to the extent of completion.

	The extent of completion				p-value Post hoc*
	I	II	III	IV	
	(<25%)	(25%≤<50%)	(50%≤<75%)	(≥75%)	
Motivational scores					
Attention	3.77±0.60	3.92±0.56	4.14±0.48	4.24±0.48	*0*.*028*
					Ⅱ<Ⅳ
Relevance	3.78±0.60	4.33±0.46	4.37±0.49	4.63±0.38	*<0*.*001*
					Ⅰ <Ⅲ
					(Ⅰ,Ⅱ,Ⅲ)<Ⅳ
Confidence	3.03±0.36	3.50±0.36	3.61±0.47	3.79±0.42	*0*.*001*
					(Ⅰ,Ⅱ)<Ⅳ
Satisfaction	3.74±0.67	3.79±0.54	4.08±0.45	4.22±0.42	*0*.*001*
					Ⅱ<(Ⅲ,Ⅳ)
Total score	3.59±0.47	3.90±0.35	4.06±0.36	4.23±0.34	*<0*.*001*
					Ⅰ <Ⅲ
					(Ⅰ,Ⅱ)<Ⅳ
Written examination score	44.26±13.27	52.40±12.33	57.46±13.61	59.31±14.73	*0*.*037*

*by Tukey test

Unit: mean±SD

After undertaking the flipped learning approach, the students stated that, by incorporating visual images before the actual sessions, the method effectively enhanced the learning process, especially in areas where conveying information solely through text is limited. Participants noted that this approach acted as a reliable guide, providing clear instructions on how to approach the sessions, including details like incision sites and direction. The preliminary video-based learning also served as an effective review tool through subsequent quizzes, reinforcing acquired knowledge and aiding retention. Consequently, students reported improved time management and reduced confusion during the dissection sessions. The utilisation of visual aids also facilitated the identification of anatomical structures without the risk of getting lost or causing damage. Another significant advantage was the promotion of independent practice, reducing reliance on practice group tutors. Regarding areas for improvement, the students noted discrepancies between the state or structure of the cadaver shown in the video and the actual cadaver used in dissection sessions. Some professional techniques and practice tools demonstrated in the video differed from the reality of the sessions. Participants also provided mixed feedback regarding the video durations. Some found it challenging to digest the amount of content presented and expressed difficulty with developing a thorough understanding of the vast material. Conversely, some participants expressed the desire to have access to the omitted intermediate process videos, suggesting that it would enhance their learning experience. Additionally, there were technical issues with video playback, resulting in interrupted viewing experiences (Table 3).

**Table 3:** Students’ feedback about the advantages and disadvantages of the flipped learning method.

Category	No (%)	Representative quotation
Advantages		
Guide to the dissection	98 (69.0%)	‘I was able to understand the purpose and draw the entire practice process in my head.’
		‘Through the video, I was able to understand the incision method and proceed with the dissection systematically.’
Self-directed learning	49 (34.5%)	‘It was good to figure out the location of the structure and its relationship with the surroundings through the video.’
		‘I watched the video more focused because of the quiz, and I could remember it longer.’
Less embarrassment	33 (23.2%)	‘We were able to start the incision without much fuss.’
Less error	14 (9.9%)	‘We were able to figure out in advance where we should be careful not to damage the structure.’
Time management	9 (6.3%)	‘It was easier to find the structure, so the time was shortened.’
Independence of tutor	7 (4.9%)	‘After flipped learning, I was able to lead the dissection independently without relying on the tutor anymore.’
Disadvantages		
Gap with reality	47 (33.1%)	‘The condition of the cadaver, dissection techniques, and practice tools in the video were different from the actual one.’
Video length and content	15 (0.6%)	‘The content of the video was so vast that it was not possible to apply all of them to actual dissection.’
		‘In the process of dissecting difficult structures, some processes were omitted. I hope the video will be supplemented.’
Video playback problem	12 (8.5%)	‘It was a pity that playback was not technically smooth when watching a video in a mobile environment.’
Less validity of quiz	11 (7.7%)	‘Difficult quizzes with overly trivial content sometimes gave me a sense of despair.’

## Discussion

The present study affirmed the efficacy of flipped learning in medical education, particularly for courses demanding high levels of professional performance from students, such as human gross anatomy dissection. This study revealed that students who used the flipped classroom for cadaveric dissection–based learning demonstrated higher attention scores than students in the traditional classroom. These findings show that relevant multimedia content—for example, images or videos in e-Anatomy^Ⓡ^—consumed during the pre-class sessions in flipped learning can play an important role in stimulating student attention [3]. In terms of a motivational design approach like the ARCS model used in this study [17], online learning content should be aligned with the four dimensions to enhance student motivation and academic achievement [18]. Although learning in the flipped classroom may not yield crucial discrepancies in terms of relatedness with students’ goals, competence in performance, and satisfaction with achievement, online content facilitated students’ interest and curiosity. Hence, to effectively deliver the flipped learning process in medical education, it is important to understand the four key dimensions (e.g., attention, relevance, confidence, and satisfaction) before designing course content.

This study also investigated the extent to which completion rates of the e-Anatomy^Ⓡ^ learning materials in the flipped classroom affected students’ motivation levels and learning achievement. The analyses showed that students who completed more of the material tended to report higher levels of motivation across the four different dimensions and demonstrated better learning outcomes. These findings appear to be consistent with the findings from Yoon *et al*.’s study, wherein the self-regulated learning group showed higher video completion rates than the non self-regulated learning group [19]. That is, students’ rigorous self-regulated learning in the pre-class sessions implies active behavioural engagement (e.g., video completion), which could lead to enhanced academic performance [19]. During the COVID-19 pandemic, a nationwide survey in Korea found that medical schools transitioned to exclusively using e-Anatomy^Ⓡ^ videos or adopted a hybrid approach incorporating online e-Anatomy during the semester, followed by hands-on dissection later [20]. This shift to a de facto flipped classroom model in the blended learning scenario was associated with superior educational outcomes when compared with the traditional lecture-based approach prior to the pandemic [20]. Likewise, in the present study, students would have been more focused on the e-Anatomy^Ⓡ^ videos to facilitate their performance and understanding of critical topics in the anatomy dissection course. Consequently, these findings show that learning through the flipped classroom before an in-person anatomy dissection course could provide students with more autonomy and self-regulated learning opportunities, affecting their motivation and academic achievement.

Additionally, this study affirmed that students’ qualitative feedback will be conducive to developing effective flipped learning courses, as flipped learning is multifaceted and can be applied to variations in practice in medical education [21]. Among the numerous benefits of flipped learning that students reported, the pre-class sessions including precise instructional videos helped students prepare for dissection sessions, which enabled them to foster confidence and reduce mistakes during in-person anatomy dissection sessions. On the other hand, content quality and technical issues—such as visually contrasting cadaver imagery, lengthy instructional videos, and compatibility issues on mobile devices—may hamper their learning experiences. Quality content and a robust platform are vital for facilitating learner–content interaction, which serves as a cornerstone for further engagement with instructors and peers [22]. Therefore, instructors should critically assess and refine content in light of student feedback to ensure it is optimally tailored to stimulate active learning [23]. Overall, this study extended the findings of Fleagle *et al*., who found that pre-class sessions associated with the flipped classroom were useful for retaining knowledge about anatomy and dissection before the in-class anatomy dissection activities [12]. This study also argued that qualitative feedback from students should be taken into account to promote their motivation and further enhance learning experiences by ultimately designing and creating coherent flipped learning content for gross anatomy dissection courses [5, 21].

Given that flipped learning may become a widely used and powerful learning method, the present study provides crucial insights into how this approach should be designed and incorporated for both students and instructors of human gross anatomy. Importantly, the flipped classroom offers foundational functions for students in the physical classroom when pre-class sessions with computer-mediated instruction are designed effectively [3]. As such, to ensure the effectiveness of flipped learning, instructors should exploit the key principles of video-based learning that make learning effective by providing students with new learning opportunities anytime and anywhere [24]. Furthermore, the findings of this study contribute to the extension of the relevant literature in which researchers have revealed the impacts of flipped learning incorporating video-based learning on student motivation [25], successful learning experiences [26], and positive student attitudes [27]. Although this study emphasised the importance of flipped learning regarding student motivation and learning achievement, we suggest that instructors consider interactivity between tutors and tutees during in-person anatomy dissection sessions. In this sense, students are likely to improve their motivation and learning efficiency because flipped learning affords students with peer support and instructor guidance [18, 28]. On the basis of the feedback from students, this study will enhance not only the quality of flipped learning but also in-class learning during human gross anatomy courses.

This study had some limitations. One of the main limitations was that this study was conducted in a semi-experimental research setting. If well-designed experimental research is carried out and includes experimental and control groups, the effects of flipped learning can be thoroughly examined. Another limitation was that we could not accurately measure how long the students concentrate although we tried to measure the extent of completion in the flipped learning by computer-generated data, such as log datasets from LMS_._ A final limitation was that customised instructional tutorials and videos were not available for our students in the gross anatomy dissection course. Faculty should develop instructional videos that are well-suited to the anatomy curriculum and course objectives based on the four dimensions of the ARCS model.

Throughout this study, we concluded that the application of flipped learning to this dissection course increased individual learning motivation, which improved in-class and out-of-class learning activities as well as academic achievement. These insights will contribute to designing effective teaching and learning strategies for gross anatomy courses and offer a novel perspective on integrating flipped learning methodologies into dissection practice. It will be necessary to track the effectiveness of flipped learning using quantitative research approaches, such as motivation scores and academic achievement, as well as qualitative research approaches, such as students’ responses.

## References

[1] Bishop J, Verleger MA, editors. The flipped classroom: A survey of the research. 2013 ASEE Annual Conference & Exposition; 2013.

[2] Moffett J. Twelve tips for “flipping” the classroom. Med Teach. 2015;37(4):331–6. doi: 10.3109/0142159X.2014.943710 25154646

[3] Persky AM, McLaughlin JE. The flipped classroom–from theory to practice in health professional education. Am J Pharm Educ. 2017;81(6). doi: 10.5688/ajpe816118 28970619 PMC5607728

[4] Sharma N, Lau C, Doherty I, Harbutt D. How we flipped the medical classroom. Med Teach. 2015;37(4):327–30. doi: 10.3109/0142159X.2014.923821 24934251

[5] McLaughlin JE, Roth MT, Glatt DM, Gharkholonarehe N, Davidson CA, Griffin LM, et al. The flipped classroom: a course redesign to foster learning and engagement in a health professions school. Acad Med. 2014;89(2):236–43. doi: 10.1097/ACM.0000000000000086 24270916

[6] Prince M. Does active learning work? A review of the research. J Eng Educ. 2004;93(3):223–31.

[7] Abeysekera L, Dawson P. Motivation and cognitive load in the flipped classroom: definition, rationale and a call for research. High Educ Res Dev. 2015;34(1):1–14.

[8] Arráez‐Aybar LA, Castaño‐Collado G, Casado‐Morales MI. Dissection as a modulator of emotional attitudes and reactions of future health professionals. Med Educ. 2008;42(6):563–71. doi: 10.1111/j.1365-2923.2008.03079.x 18452515

[9] Dyer GS, Thorndike ME. Quidne mortui vivos docent? The evolving purpose of human dissection in medical education. Acad Med. 2000;75(10):969–79. doi: 10.1097/00001888-200010000-00008 11031139

[10] Coulehan JL, Williams PC, Landis D, Naser C. The first patient: Reflections and stories about the anatomy cadaver. Teach Learn Med. 1995;7(1):61–6.

[11] Snelling J, Sahai A, Ellis H. Attitudes of medical and dental students to dissection. Clin Anat. 2003;16(2):165–72. doi: 10.1002/ca.10113 12589673

[12] Fleagle TR, Borcherding NC, Harris J, Hoffmann DS. Application of flipped classroom pedagogy to the human gross anatomy laboratory: Student preferences and learning outcomes. Anat Sci Educ. 2018;11(4):385–96. doi: 10.1002/ase.1755 29283505 PMC6381391

[13] Han E-R, Chung E-K, Nam K-I. Peer-assisted learning in a gross anatomy dissection course. PLoS One. 2015;10(11):e0142988. doi: 10.1371/journal.pone.0142988 26565616 PMC4643929

[14] Topping KJ. The effectiveness of peer tutoring in further and higher education: A typology and review of the literature. Higher Educ. 1996;32(3):321–45. doi: 10.1007/BF00138870

[15] Yoo H, Kim D, Lee Y-M. Adaptations in anatomy education during COVID-19. J Korean Med Sci. 2021;36(1). doi: 10.3346/jkms.2021.36.e13 33398947 PMC7781853

[16] Guay F, Ratelle CF, Chanal J. Optimal learning in optimal contexts: The role of self-determination in education. Can Psychol. 2008;49(3):233.

[17] Keller JM. Motivational design research and development. New York: Springer; 2010.

[18] Ma L, Lee CS. Evaluating the effectiveness of blended learning using the ARCS model. J Comput Assist Learn. 2021;37(5):1397–408.

[19] Yoon M, Hill J, Kim D. Designing supports for promoting self-regulated learning in the flipped classroom. J Comput High Educ. 2021;33:398–418.

[20] Young-Mee L, Park H. Medical education adaptation in South Korea during the COVID-19 pandemic. Asia Pac Sch. 2021;6(3):10.

[21] Chowdhury TA, Khan H, Druce MR, Drake WM, Rajakariar R, Thuraisingham R, et al. Flipped learning: Turning medical education upside down. Future Healthc J. 2019;6(3):192. doi: 10.7861/fhj.2018-0017 31660525 PMC6798025

[22] Lin G-Y, Wang Y-S, Lee YN. Investigating factors affecting learning satisfaction and perceived learning in flipped classrooms: the mediating effect of interaction. Interact Learn Environ. 2023;31(9):5759–80.

[23] Liu L, Ripley D, Lee A. Flipped learning and influential factors: Case analysis. J Educ Tech Dev Exch. 2016;9(2):5.

[24] Sablić M, Mirosavljević A, Škugor A. Video-based learning (VBL)—past, present and future: An overview of the research published from 2008 to 2019. Technol Knowl Learn. 2021;26(4):1061–77.

[25] Wang YH. Could a mobile‐assisted learning system support flipped classrooms for classical Chinese learning? J Comput Assist Learn. 2016;32(5):391–415.

[26] Al‐Zahrani AM. From passive to active: The impact of the flipped classroom through social learning platforms on higher education students’ creative thinking. Br J Educ Technol. 2015;46(6):1133–48.

[27] Akçayır G, Akçayır M. The flipped classroom: A review of its advantages and challenges. Comput Educ. 2018;126:334–45.

[28] Cocquyt C, Zhu C, Diep AN, De Greef M, Vanwing T. Examining the role of learning support in blended learning for adults’ social inclusion and social capital. Comput Educ. 2019;142:103610.

